# Dendritic Hold and Read: A Gated Mechanism for Short Term Information Storage and Retrieval

**DOI:** 10.1371/journal.pone.0037542

**Published:** 2012-05-22

**Authors:** Mariton D. Santos, Michael H. Mohammadi, Sunggu Yang, Conrad W. Liang, Joseph P. Y. Kao, Bradley E. Alger, Scott M. Thompson, Cha-Min Tang

**Affiliations:** 1 Departments of Neurology, University of Maryland School of Medicine, Baltimore, Maryland, United States of America; 2 Department of Physiology, University of Maryland School of Medicine, Baltimore, Maryland, United States of America; 3 Baltimore Veterans Administration Medical Center, Baltimore, Maryland, United States of America; Louisiana State University Health Sciences Center, United States of America

## Abstract

Two contrasting theories have been proposed to explain the mechanistic basis of short term memory. One theory posits that short term memory is represented by persistent neural activity supported by reverberating feedback networks. An alternate, more recent theory posits that short term memory can be supported by feedforward networks. While feedback driven memory can be implemented by well described mechanisms of synaptic plasticity, little is known of possible molecular and cellular mechanisms that can implement feedforward driven memory. Here we report such a mechanism in which the memory trace exists in the form of glutamate-bound but Mg^2+^-blocked NMDA receptors on the thin terminal dendrites of CA1 pyramidal neurons. Because glutamate dissociates from subsets of NMDA receptors very slowly, excitatory synaptic transmission can leave a silent residual trace that outlasts the electrical activity by hundreds of milliseconds. Read-out of the memory trace is possible if a critical level of these bound-but-blocked receptors accumulates on a dendritic branch that will allow these quasi-stable receptors to sustain a regenerative depolarization when triggered by an independent gating signal. This process is referred to here as dendritic hold and read (DHR). Because the read-out of the input is not dependent on repetition of the input and information flows in a single-pass manner, DHR can potentially support a feedforward memory architecture.

## Introduction

**Figure 1:**
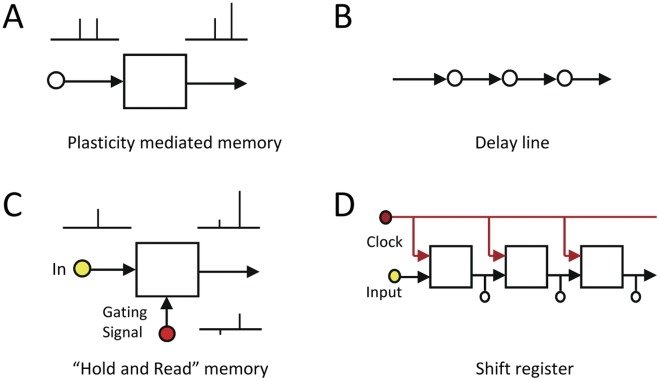
Feedforward memory architecture and it building blocks. (A) Plasticity mediated memory mechanisms such as paired pulse facilitation is better suited for feedback rather than feedforward memory, because they require repeating inputs. (B) The delay line is the simplest scheme for a feedforward memory network. Each element accepts the value of its upstream neighbor and transfers its current value to its downstream neighbor. When repeated iteratively the network transforms a time sequence into a spatial sequence. It is a memory mechanism because the spatial sequence accurately holds information on events that occurred in the past. (C) Crucial requirements for the building block of an effective feedforward memory are that each element be able to hold an input signal for a substantial length of time, and that the signal can then be read out without the signal being repeated. “Dendritic Hold and Read” (DHR), described here, can implement these requirements on individual dendrites. (D) The ‘shift register’ is the digital implementation of the delay line. It is used as the memory buffer at the input stage of the central processor unit (CPU) in digital computers. In this case the individual information holding element is the binary flipflop. It is a discrete time network because information is binary and is moved forward at set time intervals by a clock. The entire spatial sequence can be accessed by tapping the output of each of the flipflops simultaneously.

Processing of time-encoded information requires memory. Without some form of short term memory buffer to hold together a temporal sequence, the individual bits of information that are received at any moment in time cannot be properly interpreted. For example, in order to predict the trajectory of a flying ball, it is necessary to hold in mind a temporal sequence of recent positions of the ball. Similarly, the meaning of a sentence can be dramatically different depending on the order of the words and the presence or absence of a single word. The cellular mechanism for such a short term memory buffer is poorly understood. Discussions in the past on the cellular basis of working memory [1], [2] and, by inference, short term memory, have not considered how the nature of the memory content may constrain the memory architecture and vice versa. Recently, some in the neural computational field began to question whether the memory networks proposed to handle static signals can adequately handle dynamic signals [3]–[5]. Current models postulate that the memory trace is maintained by synaptic reverberations within a recurrent feedback network [1], [2]. Mechanisms of synaptic plasticity such as paired pulse facilitation (PPF) (Figure 1A) and NMDA-stabilized recurrent synapses have been proposed to establish ‘attractor’ states [6], [7]. But ‘attractor’ states are poorly suited to hold time-varying signals [3], [5]. This has led to the idea that dynamic signals may utilize feedforward rather than feedback memory architecture [3]–[5], [8]. One abstract way for imagining a feedforward memory system is by analogy with the way in which the propagation of ripples in a pool of water can hold information on where and when pebbles were dropped into the pool in the recent past [5]. The property of the pool of water does not change in order to hold the input information. In contrast, an ‘attractor’ state in a feedback memory system is analogous to a rapidly adapting resonant chamber. The resonant property of such a chamber or network has to constantly adapt and change in response to its most recent input. This is two fundamentally distinct strategies for holding information with significant functional implications. The question that motivated this study is whether the nervous system possesses the molecular and cellular mechanisms that can support a feedforward memory architecture. Here we show that such mechanisms do exist and is widely present on hippocampal pyramidal neurons. Preliminary findings have been presented in abstract form [9]–[11].

The simplest form of a feedforward memory mechanism is the delay line (Figure 1B). It consists of a linear chain of identical elements connected in series. A requirement of each element in the chain is that it be able to hold an input signal for a finite period of time, and then afterwards send that signal to its downstream neighbor (Figure 1C). Such a network can hold and read-out inputs that are sequences in time. The first element takes on the state of the input arriving at that moment in time. When the next bit of input information arrives, the first element transfers its state to its downstream neighbor and it, in turn, receives the state of the new input. With repetition an entire temporal sequence can be accepted and held in the linear chain. By tapping all of the elements simultaneously the entire sequence that arrived over a period of time can be accessed as a whole. In addition to the memory function, this process also implements a transformation from temporal pattern to spatial pattern. Spatial patterns can be efficiently processed by recurrent attractor networks. Their implementation in the form of the ‘shift register’ at the input stage of the CPU in every digital computer (Figure 1D) provides ample evidence that delay line memory is more than just a theory. The shift register is a discrete-time, feedforward-memory buffer for the CPU. The signal holding element in the shift register is the binary flipflop that can switch between one of two states. The state of the flipflop is determined by the state of the input when the clock signal arrives. While the computer and the brain process information in markedly differing manners, they both need short term memory buffers since they both receive information that arrive stretched out over finite periods of time.

There were two reasons why delay line memory had been dismissed as a memory buffer mechanism in the nervous system. First, plausible biological mechanisms for mediating time delays longer than a few milliseconds had not been identified. The only physiological mechanism considered was the short delay involved with the propagation of an action potential. Delays of a few milliseconds would not provide memory of meaningful duration. Second, without some means to maintain the signal-to-noise ratio, the memory trace would degrade rapidly as the signal was passed from element to element in the linear chain. This is not a problem in the shift register because the flipflop that constitutes the building block for the shift register is a binary device and digital transmission resists signal-to-noise degradation. However, no such analogous feature was known to exist at the level of dendritic integration. Here we propose and demonstrate a new neurophysiological mechanism on dendrites, referred to here as “dendritic hold and read” or DHR, that has the potential to satisfy these two requirements for a biological delay line. We postulate (a) that a memory trace exists in the form of patterns of NMDA receptors in the ligand-bound but Mg^2+^-blocked state [12], (b) that information read-out takes the form of local regenerative spikes driven by state transitions of these quasi-stable NMDA receptors, and (c) that conditional read-out is gated by a depolarization that is independent of the input. Because the bound-but-blocked state of the NMDA receptor is a long-lived state determined by the slow unbinding of glutamate from the receptor, it has the potential to provide delays of hundreds of milliseconds. And because the information read-out is a regenerative dendritic spike, it is effectively an amplified binary output that resists degradation by noise during propagation.

The dendritic hold and read (DHR) hypothesis provides testable predictions. Whereas NMDA receptors in the reverberating feedback model are postulated to be in their conducting state [7], NMDA receptors when holding information in DHR, would be in their non-conducting state. Therefore, one would predict that during the period of time between the original stimulus and the read-out response, the dendrite can be electrically silent. Paired pulse facilitation (PPF), a mechanism previously proposed to support feedback mechanism [6] is similar to DHR in that they operate in the same time regime. PPF is characterized by graded potentiation, whereas DHR should display all-or-none behavior. Therefore, if short term memory is implemented by DHR rather than a PPF like mechanism, a second prediction is that the read-out of the information will be binary rather than graded in nature. In plasticity mediated (Figure 1A) and reverberating feedback mechanisms the input signal must be repeated from the same synapses to initiate read-out, whereas in DHR a gating signal from a separate set of synapses initiates read-out (Figure 1C). Therefore, a third prediction is that the information that is stored by DHR can be retrieved without its being repeated over the identical input lines.

## Results

**Figure 2:**
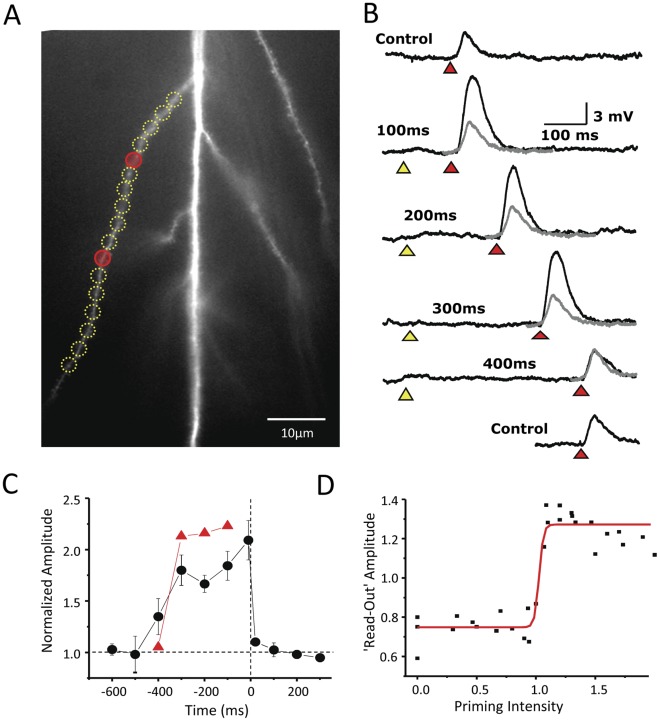
The dendritic hold and read hypothesis. (A) In the ‘dendritic hold and read’ hypothesis, information is held by the ligand-bound but Mg2+-blocked state of the NMDA receptor. To create such a population of receptors, a length of radial oblique dendrite is ‘primed’ by a low concentration of photoreleased glutamate (yellow circles). The read-out of the priming signal is postulated to be triggered by a modest ‘gating’ depolarization. This gating signal is generated by stronger focal photolysis of glutamate to activate AMPA receptors (red signal). (B) The priming stimulus need not produce any depolarization (yellow triangle). The gating stimulus (red triangle), when given in isolation, produces a 3–5 mV somatic depolarization (Control). But if the priming and gating stimuli are paired within a certain window of time (in this case ≤300 ms), an enhanced depolarization above the control response is observed (black vs. gray traces). The response to the gating stimulus when paired to the priming stimulus is called the ‘read-out’ response. (C) The ‘read-out’ response can be expressed as a function of the relative timing of priming and gating signal. The normalized response of the dendrite illustrated in panel B is shown in red. Group data for 42 dendrites is shown in black. (D) The amplitudes of ‘read-out’ responses as a function of priming intensity show a step-like behavior, also consistent with the idea that the read-out response is a local dendritic spike.

**Figure 3:**
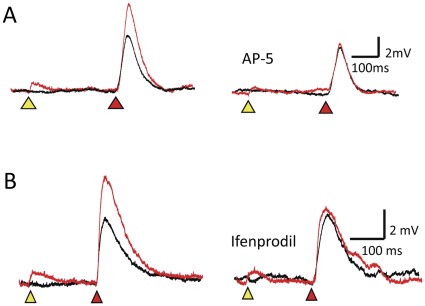
NMDA receptors mediate DHR. (A) The potentiation associated with DHR at an ISI of 300 ms (left) is completely eliminated after application of AP-5 (right). (B) The potentiation associated with DHR at an ISI of 200 ms (left) is also attenuated after application of the NR2B subunit selective antagonist, ifenprodil (right).

One testable prediction of the DHR hypothesis is that the NMDA receptors and the dendrite on which they are located can be electrically silent during information storage. To test this prediction, photolysis is used to release a low concentration of free glutamate that is sufficient to bind to the high affinity NMDA receptors over a restricted region of a terminal dendrite, (yellow circles in Figure 2A) but below that needed to activate the low affinity AMPA receptors. A small degree of AMPA-dependent depolarization could sometimes be observed immediately following photolysis. This transient depolarization was insensitive to NMDA receptor antagonists (Figure 3) and lasted <100 ms. Without strong AMPA receptor activation there will be insufficient membrane depolarization to reverse the Mg^2+^ blockade of the NMDA receptors. As a result most of the NMDA receptors would enter into the long-lived, non-conducting, bound-but-blocked state. Indeed, in response to an initial low intensity photolytic release of glutamate, little to no depolarization is observed (marked by yellow triangles in Figure 2B). This is referred to here as the priming stimulus. The objective in using the priming stimulus is to create a high level of NMDA receptors in this quasi-stable state within a single dendritic electrical compartment.

With our photostimulation system, laser pulses can be targeted to multiple independent sites along a dendrite. Hence, we can deliver a priming stimulus to one set of locations, and a gating stimulus to a separate set of locations. For example, Figure 2A illustrates laser pulses directed at gating sites (red circles) distinct from the priming sites (yellow circles) independently applied to the same dendrite. The laser energy to the gating sites was adjusted to evoke a 3–4 mV depolarization recorded at the cell soma (red triangle, Figure 2B). This stimulus is referred to here as the “gating” signal. The responses to the gating signal in the absence of the priming stimulus are labeled as control in Figure 2. If the priming input is coupled to the gating signal within a certain time window, in this case ≤300 ms, then the response is markedly potentiated relative to the control response (Figure 2B). The response to the gating stimulus coupled to the priming stimulus is referred to here as the “read-out” response. The enhancement of the read-out response is not due to classical temporal integration since the experimental conditions were set up to ensure that the membrane was not depolarized at the time of the second pulse. To verify that this potentiation is indeed mediated by NMDA receptors experiments were conducted before and after application of the NMDA antagonist, AP-5 (100 µM). There is complete block of the potentiation in each case (n = 6) (Figure 3A). To determine whether NMDA receptors containing NR2B subunits participate in this form of potentiation, experiments were conducted with and without the NR2B selective antagonist, Ifenprodil (1–2 µM). The potentiation was reduced by 79±4% (n = 3) (Figure 3B). Potentiation in the presence of another NR2B selective antagonist, Ro-256981 (0.5–1 µM) was reduced by 84±11% (n = 6). Because the amplitude of the read-out response is linked to the recent history of glutamate exposure, we propose that this phenomenon may support an elementary form of short term memory. And because little to no depolarization is observed during the period between the priming stimulus and the read-out response, these observations support the prediction that short term memory can be held silently by the bound but blocked NMDA receptors. The electrical silence of short term information storage has profound implications for memory capacity and energy consumption.

DHR is superficially similar to paired pulse facilitation (PPF) in some respects, but unlike classical PPF, which reflects changes in the presynaptic terminal, DHR is exclusively a postsynaptic phenomenon. However, a recent description of a post-synaptic variant of PPF would appear to blur the differences between DHR and PPF [13]. One characteristic that can distinguish between PPF and DHR is whether their respective potentiation is a graded or an all-or-none phenomenon. PPF has been consistently shown to be a graded phenomenon that is a function of both the interstimulus interval (ISI) between the two stimulus pulses and the relative strength of the pulses [14]. As noted earlier, the read-out response accompanying DHR is an all-or-none NMDA spike. The DHR hypothesis predicts that the potentiation observed in response to the DHR experimental paradigm will be a binary event. This prediction is tested in the experiment shown in Figure 2D. Note that the amplitude of the potentiated read-out responses are nearly identical at ISI between 100 and 300 ms. But if the ISI is increased slightly to 400 ms, the potentiation is absent. This binary behavior is due to the proximity of the threshold for regenerative depolarization and the threshold for response saturation. Response saturation takes place when the dendritic membrane depolarizes towards the reversal potential of the NMDARs. The low threshold for response saturation is because of the high input impedance of the thin terminal dendrites. While individual dendrites reliably demonstrate this binary behavior, when the time-dependence of DHR on 42 separate dendrites are averaged the function appears to be graded (black circles, Figure 2C). However, there is considerable variability in the ISI dependence between different dendrites, and it is likely that variability in timing, rather than the responses themselves accounts for this appearance. A similar all-or-none behavior can be observed at the level of individual dendrites when the amplitude of the read-out response is plotted as a function of the intensity of the priming stimulus. At very low priming intensity when glutamate is photoreleased at a level below the threshold for NMDA spike initiation, there is no observable potential. As the priming intensity is gradually increased, the read-out response abruptly switches to a saturating response (Figure 2D). The all-or-none characteristic of the read-out response to the DHR experimental paradigm supports the notion that our protocol has uncovered a new phenomenon, DHR, that is distinct from PPF. The binary nature of DHR has important implications for the ability to maintain the fidelity of a memory trace as it is propagated in time.

**Figure 4:**
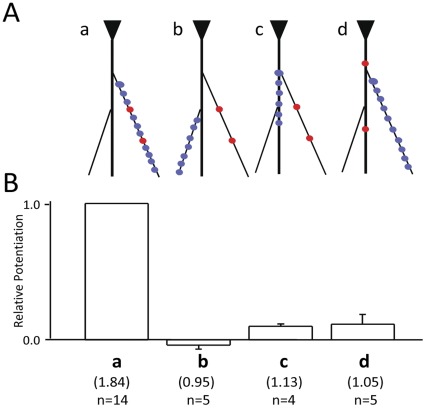
DHR is a compartmentalized phenomenon. (A) Positive DHR responses occur only when the priming (blue circles) and gating (red circles) stimuli arrive within the same individual dendritic electrical compartment (configuration a). (B) Other configuration (b–d) fail to show potentiation with comparable stimulation intensities in the same cell. Statistics of the potentiation in each configuration is provided.

Another difference between DHR and PPF is the morphologic substrate for their elementary unit of excitability. For PPF it is individual synapses. For DHR it is functional electrical compartments on the dendritic arbor. Electrical compartmentalization is determined by a combination of factors such as dendritic morphology, active intrinsic conductances, and the pattern of synaptic inputs. Assuming a diffuse pattern of synaptic excitation, the input impedance mismatch at the junction of the thin terminal dendrites to the thick apical trunk, would suggest that the compartments are likely to comprise individual terminal dendrites [15]. To test this prediction, different spatial patterns of priming stimuli were coupled to different gating stimuli (Figure 4). Potentiation could only be observed when the priming and the gating stimuli were co-localized on the same terminal dendrite (Figure 4B).

**Figure 5:**
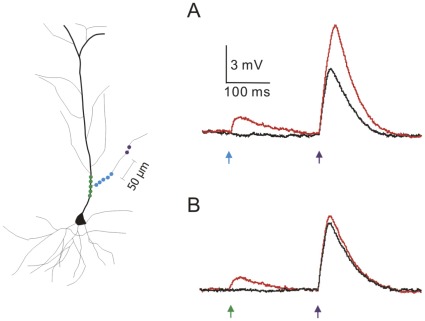
A distinction between DHR and PPF. (A) When the priming stimulus (blue circles) and the gating stimulus (purple circles) are separated by >50 µm, potentiation can still be observed. This separation makes it unlikely that the same set of NMDA receptors could be activated by the two stimuli. (B) As an internal control, when the priming stimulus is directed on the adjacent apical trunk (green circles) potentiation is not observed.

The most important characteristic of DHR from the perspective of memory architecture is that it does not require the stored information to be repeated identically in order for it to be read-out. Synaptic plasticity mediated mechanisms such as PPF (Figure 1A), in contrast, do require such repetition. While repetition is well suited for reverberating-feedback types of memory architectures, it is not compatible for feedforward architecture in which information is propagated in time in a single pass manner (Figure 1B-D). Because of this fundamental implication, additional steps were taken to distinguish between DHR and PPF. A concern is that glutamate photoreleased during the priming and gating stimuli might have reached a shared set of receptors on a given dendritic branch via lateral diffusion, in which case what appeared to be DHR, would actually be a variant of post-synaptic PPF [13]. If that were the case, then potentiation would not be observed if the two stimuli were separated widely in space. To test this prediction, the experimental protocols illustrated in Figure 2 are repeated with the exception that the priming and gating stimuli are targeted to opposite ends of the long oblique dendrites and are separated by at least 50 µm (Figure 5A). Diffusion of an already low concentration glutamate to a location over 50 µm away in the presence of avid glutamate transporters will not yield measurable levels of glutamate. Potentiation can still be consistently observed when the input and gating signals are made clearly distinct in terms of their source and target. As a further control for the diffusion of glutamate and as a test of the prediction that the morphologic substrate for DHR is the thin terminal dendrites, the priming stimulus was directed on the adjacent main apical trunk (Figure 5B, green circles and arrow). Potentiation could not be observed in this case. Thus, the potentiation observed here represents a novel cellular mechanism of response potentiation and not a form of dendritic PPF.

## Discussion

In summary, these experiments demonstrate that synaptic activity, as simulated with photoreleased glutamate, can leave a transient “memory trace” on individual dendritic branches of pyramidal neurons. This memory trace can persist up to hundreds of milliseconds in the form of bound-but-blocked NMDA receptors and can be conditionally retrieved by an independent gating depolarization. Read-out of the information is in the form of all-or-none local regenerative spikes. DHR represents a new form of information storage and retrieval. We propose that DHR constitutes an elementary building block for feedforward memory.

The experiments described here represent the first report of DHR, and accordingly, simply reveals its essential properties. One of the goals was to determine whether the bound-but-blocked NMDA receptors are necessary and sufficient to hold information in DHR. This question could be most clearly demonstrated by minimizing AMPA receptor activation during the priming stimulus. However, it will be important in future studies to demonstrate DHR under conditions of AMPA receptor activation. We know that modest increases in AMPA receptor activation do not prevent potentiation by DHR (Figure 5A). Strong increases in AMPA receptor activation was avoided here to prevent overlap between the priming depolarization and the read-out response, a condition that would complicate the distinction between DHR and temporal integration. In order to more precisely quantitate the ISI time dependence of DHR, the priming stimulus was provided as an instantaneous spatially distributed input, rather than a temporally dispersed or continuous background synaptic excitation. Photolytic release of glutamate enabled us to administer priming and gating stimuli with great precision. It will of course ultimately be necessary to replicate these observations with selective stimulation of individual glutamate synapses. The nature of the gating stimulus is still unclear. It need not be a localized AMPA mediated depolarization. The only requirement for the gating stimulus is that it is able to generate a modest membrane depolarization on the terminal dendrite. In order to demonstrate the participation of NR2B-subunit-containing NMDA receptors the experiments were conducted at ISI up to 300 ms. There is no reason to expect that NR2A-subunit-containing receptors would not participate in DHR at shorter ISIs.

**Figure 6:**
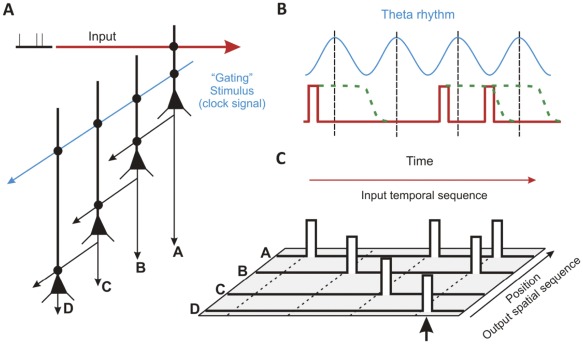
DHR as the elementary building block for a feedforward memory architecture. (A) The schematic illustrates one simple configuration of four hippocampal pyramidal neurons capable of temporal sequence recall. A sequence of three irregular pulses is modeled as the input (red) to a dendritic branch on cell A. A gating signal (blue), possibly a component of the theta rhythm, is a separate input onto the same dendrite. (B) If the input primes sufficient numbers of NMDARs within a time window (green dashed line) before the peak of the theta rhythm, a dendritic spike is generated which greatly increases the probability that cell A fires an action potential that is sent as input to cell B. (C) After four cycles the temporal sequence in this example is transformed into a spatial sequence encoded by activity in four adjacent neurons. Such a spatial pattern can then be recognized by classic attractor networks.

A diagram illustrates the essential features of a DHR-based network mechanism (Figure 6). Experimental validation of the proposed network will be the subject of subsequent studies. The neurons in the chain are functionally identical and are unidirectionally connected. A rhythmic oscillation, such as theta rhythm, could serve as a gating signal. The theta frequency is compatible with the off-rate of NR2B containing NMDA receptors [16] and the observed duration for DHR (Figure 2B and 2C). The input, comparable to the priming stimulus in our experiments, would consist of action potentials arising from multiple neurons firing in a rate-encoded manner in response to a common stimulus. The inputs would arrive on one or more oblique or basal dendrites of pyramidal neurons. When local spikes, comparable to the read-out response, are generated from multiple terminal dendrites, they would drive the neuron to fire a somatic action potential [11]. The serial network shown in Figure 6A is an abstract version of multiple parallel connections. Such a parallel connected scheme could answer the criticism that a simple delay line would be vulnerable to disruption if a single element is lost.

The proposed scheme illustrated in Figure 6 brings up a number of salient issues. Recent proposals for feedforward memory networks have shied away from discrete time models (i.e. models with clocks) [3]–[5], [8]. But if information in the brain is encoded in a spike rate manner, then fidelity of the information must be integrated over fixed time windows. Moreover, theta rhythms are likely to serve vital functions for learning and memory [17]. Alternatively, it has been suggested that the backpropagating action potential (bAP) could serve as the gating signal. But a gating stimulus arising proximal to the more distal location of a priming stimulus, as would be the case with bAP, is a poor location to produce potentiation in DHR. In this case, bAPs create significant decreases in membrane resistance that shunt the current needed to sustain the regenerative depolarization of the DHR read-out mechanism. In fact, we observe that the most effective means for eliciting DHR is for the gating stimulus to be distal to the priming stimulus as shown in Figure 5A. Furthermore, from a feedforward memory architecture perspective the best candidate gating or clock signal is one that is completely independent of the input signal. The bAP is not independent of the cell’s recent synaptic inputs.

The distinctive properties of DHR and the potential power of a feedforward architecture have significant functional implications. Because memory storage in DHR is electrically silent, the amount of energy consumed in holding information would be minimal. Energy is consumed only for the portion of information that is retrieved. This is in sharp contrast to what would be expected for reverberating-activity models of short term memory. Because DHR mediated information storage does not require repetition or forewarning, it is well suited to handle the single pass and arbitrary nature of dynamic stimuli that a nervous system must handle. Together these two properties enable the nervous system to transiently hold massive amounts of information and to perform specific retrieval tasks at later times. It is likely that both feedforward and feedback mechanisms are needed for behavior. Feedback network mediated memory would operate downstream to feedforward memory because DHR can implement two fundamental signal transformations that are needed before the information can be accepted by feedback networks. DHR mediated feedforward memory has the capacity to transform temporal sequences into spatial patterns. DHR also mediates an analogue-to-digital transformation that significantly compresses the size of the input. Individual synaptic events arriving over time are integrated in brief epochs and converted into simple binary outputs. Inputs from hundreds of synapses on a single terminal branch are also integrated as a single electrical compartment. This massive signal compression in time and space may be necessary for processing in the nervous system. It may also explain why memory, even very short term memory, can never have the resolution of a real experience. Feedback networks have their own unique powers such as the ability for plasticity and longer term storage of complex spatially distributed patterns. We speculate that a two stage system, feedback-piggybacked-to-feedforward network, would be a flexible, efficient, and powerful short term mnemonic system.

## Methods

### Methods for Patterned Photolysis

**Figure 7:**
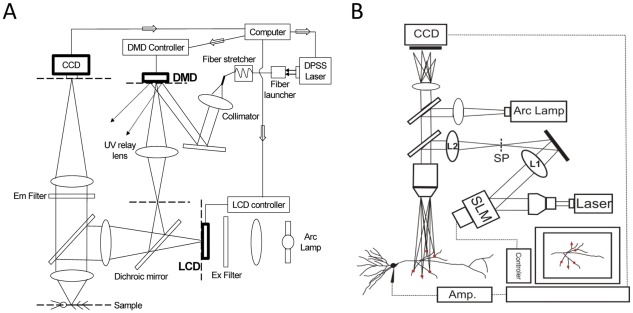
Photolysis systems. (A) The DMD system utilizes the ability of the DLP® chip from Texas Instrument for creating user specified 2D spatial patterns. These patterns, generated by the hundreds of thousands of digital micromirrors, are used to direct and project a portion of the 355 nm output of a 1 W DPSS laser onto the dendritic arbor in the acute brain slice. Different spatial patterns can be saved by the computer and projected at video rates. (B) The holographic system utilizes the capability of the phase-only spatial light modulator (SLM) from Hamamatsu to create a hologram that projects a 3D user defined pattern onto the dendritic arbor. The light energy driving the system is a 150 mW 405 nm diode laser. Different spatial patterns can be saved by the computer and projected at video rates.

Photolysis of caged glutamate provides the best means for precisely controlling the temporal and spatial pattern of stimulation necessary to test the theory of dendritic hold and read [18]. We employed either of two recently described photolysis systems [19], [20]. The first system utilizes a digital micromirror device (DMD) to created patterned photostimulation (Figure 7A). The second system utilizes a phase only spatial light modulator (SLM) to create 3D holographic photostimulation (Figure 7B). Early experiments were conducted with the DMD system and later experiments were conducted with the holographic system. Both systems are able to produce the same physiological response. However, the holographic system is more flexible and simpler to operate. A second difference is that the holographic system is able to provide diffraction limited focusing and therefore can provide faster uncaging of glutamate associated with the gating signal. The poorer focusing ability of the DMD system is secondary to bringing the UV laser beam through the epifluorescence tube lens which is optimized for illumination rather than imaging. This limitation may be potentially solved by replacing the existing tube lens with a lens designed specifically for the UV laser. This is not an issue with the holographic system because there is no illuminating tube lens. Focusing is achieved through the hologram generated by the SLM (Figure 6B). A third difference is that the holographic system is powered by a 405 nm laser and requires the use of MNI-glutamate (1–2 mM), whereas the DMD system is powered by a 355 nm laser that could use a variety of caged glutamate. Experiments carried out with the DMD system used NCM-Glu (1–2 mM).

### Caged Glutamate

Two forms of caged glutamate were used, MNI-Glu and Ncm-Glu. MNI-glutamate was purchased from Tocris or Femtonic. NcmM-Glu (*N*-[(6-nitrocoumar-7-yl)methyl]-L-glutamic acid) was synthesized in-house. Its preparation is described here. 0.9 g of 7-bromomethyl-6-nitrocoumarin (3.1 mmol; obtained by brominating 7-methyl-6-nitrocoumarin with *N*-bromosuccinimide in the presence of benzoyl peroxide), 1.04 g of H-Glu(OtBu)-OtBu·HCl (3.5 mmol), 1.4 mL of triethylamine (10 mmol) and 6 mL of dry DMSO were added. The mixture was stirred at room temperature for 4.5 h. The reaction mixture was dried under high vacuum, and the residue was purified by column chromatography (5–10% CH_3_CN/CH_2_Cl_2_) to yield *N*-[(6-nitrocoumar-7-yl)methyl]-L-glutamic acid, di-*t*-butyl ester as a viscous oil (0.83 g, 57% yield). ^1^H NMR (CDCl_3_) 8.22 (s, 1 H), 7.77 (d, 1 H, *J* = 9.5 Hz), 7.72 (s, 1 H), 6.54 (d, 1 H, *J* = 9.5 Hz), 4.14 (dd, AB type, 2 H, *J*_AB_ = 16 Hz), 3.15 (m, 1 H), 2.38 (m, 2 H), 1.98–1.82 (m, 2 H), 1.47 (s, 9 H), 1.45 (s, 9 H). For deprotection, 0.1 g of the di-*t*-butyl ester was dissolved in 1 mL glacial acetic acid; 1 mL conc. HBr was added, and the reaction mixture was maintained in an ice/water bath for 15 min. The reaction mixture was reduced under vacuum, and the residue was dissolved in 1.2 mL water and purified by reverse-phase HPLC (6∶4 acetonitrile–water, with 0.1% v/v trifluoroacetic acid). Product fractions were pooled and lyophilized to yield 0.052 g of *N*-[(6-nitrocoumar-7-yl)methyl]-L-glutamic acid, or *N*-Ncm-Glu (68% yield). ^1^H ((CD_3_)_2_SO) 8.49 (s, 1 H), 8.15 (d, 1 H, *J* = 9.8 Hz), 7.71 (s, 1 H), 6.63 (d, 1 H, *J* = 9.5 Hz), 4.05 (dd, AB type, 2 H, *J*_AB_ = 15.3 Hz), 3.17 (m, 1 H), 2.32–2.29 (m, 1 H), 1.86–1.80 (m, 1 H), 1.76–1.66 (m, 1 H). Mass spectrum (HR FAB): calculated for C_15_H_15_N_2_O_8_ (MH^+^), 351.0828; found 351.0829.

### Brain Slice Recordings

All procedures were approved by the Institutional Animal Care and Use Committee at the University of Maryland School of Medicine. Hippocampal slices were prepared from 2–3 week old Sprague-Dawley rats. Rats were deeply anesthetized and intact hippocampi were quickly removed and placed in chilled artificial cerebral spinal fluid (aCSF) containing (mM): 145 NaCl, 3 KCl, 10 HEPES, 2 CaCl_2_, 1 MgCl_2_, and 10 glucose, and were then cut into 400 uM coronal slices using a vibrating blade microtome. Slices were transferred to a holding chamber containing aCSF at room temperature which was bubbled with carbogen (95% O_2_/5% CO_2_) for at least 1 hour prior to recording. Slices were then transferred to a recording chamber and constantly perfused with carbogen-saturated aCSF. Unless otherwise stated, all experiments were performed at temperatures between 32–35°C. Whole-cell recordings were done “blind” using an Axon Instruments Axoclamp 700B Amplifier and pClamp Version 10.2 software was used for data acquisition. Glass recording pipettes (resistance 3–6 megaOhms) were filled with an internal saline solution containing (mM): 135 KCH_3_SO_3_, 10 HEPES, 10 NaCl, 1 MgCl_2_, 0.1 mM K_4_BAPTA, 2 mM Mg^2+^-ATP, and 10 mM Phosphocreatine, buffered to pH 7.3 with KOH. Alexa 594 (50–100 µM) was included in the internal solution to allow for visualization of the dendrites. Tetrodotoxin (TTX, 1 µM) was included in the aCSF for most experiments. All recordings were done in the presence of the GABA_A_ antagonist bicucuilline methiodide (10–20 µM), GABA_B_ antagonist CGP-35348 (5–20 µM), and 0.1 µM glycine. Caged glutamate (NCM-Glu, 0.5 mM) was bath applied. Following a 10 minute baseline, an I-V curve was then established to determine passive properties of the cell. Cells were discarded if R_a_ increased above 25 mOhms at any point during the experiment. Recordings were done in “current-clamp” configuration and cells were held between −65 to −73 mV. All chemicals and drugs, including ifenprodil and Ro25-6981, were obtained from Sigma-Aldrich.
